# Advancing Physiology with Expanded Multi-Omics

**DOI:** 10.1093/function/zqac031

**Published:** 2022-06-20

**Authors:** Mingyu Liang, Allen W Cowley, Andrew S Greene, Aron M Geurts, Pengyuan Liu, Yong Liu, Sridhar Rao

**Affiliations:** Center of Systems Molecular Medicine, Department of Physiology, Medical College of Wisconsin, Milwaukee, WI, 53226, USA; Center of Systems Molecular Medicine, Department of Physiology, Medical College of Wisconsin, Milwaukee, WI, 53226, USA; The Jackson Laboratory, Bar Harbor, ME, 04609, USA; Center of Systems Molecular Medicine, Department of Physiology, Medical College of Wisconsin, Milwaukee, WI, 53226, USA; Center of Systems Molecular Medicine, Department of Physiology, Medical College of Wisconsin, Milwaukee, WI, 53226, USA; Key Laboratory of Precision Medicine in Diagnosis and Monitoring Research of Zhejiang Province, Sir Run Run Shaw Hospital, Zhejiang University School of Medicine, Hangzhou, 310030, China; Cancer center, Zhejiang University, Hangzhou, 310030, China; Institute of Translational Medicine, Zhejiang University School of Medicine, Hangzhou, 310030, China; Center of Systems Molecular Medicine, Department of Physiology, Medical College of Wisconsin, Milwaukee, WI, 53226, USA; Versiti Blood Research Institute, Milwaukee, WI, 53226, USA; Department of Cell Biology, Neurobiology, and Anatomy, Medical College of Wisconsin, Milwaukee, WI, 53226, USA; Division of Pediatric Hematology-Oncology, Medical College of Wisconsin, Milwaukee, WI, 53226, USA

**Keywords:** physiology, multi-omics, genetics, epigenetics, systems biology, computational biology, deep sequencing, gene expression, kidney, blood pressure

Advances in molecular biology in the second half of the 20th century shifted the view of human biology from one that was exclusively based on organ systems to one that emphasized molecules. In the last decade or so, the view of human biology has begun to shift again, this time towards an increasing recognition that humans are molecular systems in which molecules interact to take on emergent properties within the context of cells and organ systems. The increasing recognition that humans are molecular systems has led to the emergence of the new scientific discipline of molecular systems medicine^1^.

One of the pillars of molecular systems medicine is the integration of genome-scale molecular analysis, or omics, with physiology. The idea of using omics to advance physiology is not new. What is new is the unprecedented opportunity in this area provided by the substantial expansion of omics toolbox and knowledgebase in the last 10–15 years.

The first RNA-seq paper was published 15 years ago. Since then, deep sequencing-based methods have been developed for genome-scale analysis of chromatin conformation (e.g., Hi-C and Micro-C), chromatin accessibility (e.g., assay for transposase-accessible chromatin with sequencing, or ATAC-seq), histone binding (e.g., cleavage under targets and tagmentation, or CUT&Tag), and DNA methylation (e.g., reduced representation bisulfite sequencing, or RRBS). Methods for genome-scale analysis of RNA (including RNA modifications, e.g., methylated RNA immunoprecipitation sequencing, or MeRIP-seq), small noncoding RNA, proteins, and metabolites have also continued to advance. Many of these assays can now be performed in single cells or single nuclei (i.e., single cell omics) as illustrated by recent reports of pan-tissue single-cell transcriptome atlases in humans^2^. Furthermore, several omic assays may be integrated with spatial distribution of cells in native tissues (i.e, spatial omics).

The power of multi-omics is enabled and amplified by advances in bioinformatics and the integration with computational modeling. For example, a recent study used experimental analysis and mathematical modelling to quantify the effect of long-range chromatin interactions on transcriptional activities in single cells^3^. The analysis revealed a nonlinear relation between the transcriptional effect of an enhancer and the enhancer's contact probabilities with the promoter, which might arise from the transient nature of enhancer-promoter interactions coupled with slower promoter bursting dynamics in individual cells.

Application of this ever-expanding multi-omics toolset has led to the generation of extensive multi-omics knowledgebases. For example, ENCODE (The Encyclopedia of DNA Elements), along with NIH Roadmap Epigenomics, have developed laboratory and data standards for an array of epigenomic assays and applied these assays to analyze several hundred cell lines and tissue specimens^4^. A core product of this effort is the Registry of candidate regulatory elements (cREs). The current version of the Registry contains 1310152 human cREs and 527001 mouse cREs. The GTEx (Genotype-Tissue Expression) program has established an extensive catalog of genetic variants associated with gene expression, which are called expression quantitative trait loci (eQTLs)^5^. The final dataset from GTEx (V8) contains DNA data from 838 postmortem donors and 17382 RNA-seq datasets across 54 tissue sites and two cell lines.

The rapid expansion of multi-omics toolbox and knowledgebase provides unprecedented opportunities to advance our understanding of physiology and pathophysiology as illustrated by an increasing number of studies. Gupta, et al., integrated genetic fine mapping, genome editing in stem cells, and gene expression and chromatin conformation analyses to identify and study a DNA sequence variation associated with five vascular diseases^6^. They found evidence for a long-range effect of rs9349379, a common single nucleotide polymorphism, on the expression of *EDN1* (encoding endothelin 1) located more than 600 kb away.

In a study of human kidney biopsy specimens, we developed an approach to perform small noncoding RNA deep sequencing analysis in several hundred glomerular and tubulointerstitial regions, each with a known pathological state^7^. The analysis generated pathologically resolved small RNA maps and revealed novel insights into kidney disease progression such as the presence of substantial molecular changes in patient glomeruli and tubulointerstitial regions that are histologically indistinguishable from kidney tissue regions in healthy people.

We recently performed an analysis of more than 26 000 single nucleotide polymorphisms in more than 1000 genomic loci that had been associated with human blood pressure with genome-wide significance^8^. The analysis integrated 14 data types, including eQTL data from GTEx and enhancer data from ENCODE, and incorporated a list of 251 genes that we called “blood pressure physiology genes” as they had been associated with blood pressure regulation in the literature. The analysis provided a wide range of new insights into how genes relevant to the physiology of blood pressure might be regulated at the genome level.

Physiology and multi-omics are mutually beneficial. Multi-omics provides global views of the molecular basis of physiology and can reveal unsuspected dimensions of regulatory mechanisms important for understanding physiology and linking physiology with genetics and disease. Physiology moves multi-omics from cataloging molecular parts and features and studying reductionist models to generating new insights into complex organ systems function under normal, stressed, or diseased conditions.

Studies that organically integrate multi-omics with physiology will be especially powerful. Such integration will require team science collaborations between forward-looking physiologists, genomicists, and computational biologists or, better yet, scientists who are cross-trained in physiology, multi-omics, and computational biology. The integration will benefit from precise experimental interventions made possible by advanced genome editing technologies and the development of model systems that are highly relevant to human health and disease, including animal models and human induced pluripotent stem cell models. Just like the integration of physiology and multi-omics, molecular perturbation in informative model systems is a pillar of molecular systems medicine.

Molecular systems medicine is the future of medicine and biomedicine. One must understand humans as molecular systems to truly understand human health and disease. Advancing physiology with expanded multi-omics will help to put physiology at the center of the molecular systems medicine revolution and keep it there.

## Data Availability

Not applicable.

## References

[1] Liang M. Epigenetic Mechanisms and Hypertension. Hypertension. 2018; 72(6):1244–1254. 30571238 10.1161/HYPERTENSIONAHA.118.11171 PMC6314488

[2] Liu Z , Zhang Z. Mapping cell types across human tissues. Science. 2022; 376(6594):695–696. 35549410 10.1126/science.abq2116

[3] Zuin J , Roth G, Zhan Y, Cramard J, Redolfi J, Piskadlo E, Mach P, Kryzhanovska M, Tihanyi G, Kohler H, Eder M, Leemans C, van Steensel B, Meister P, Smallwood S, Giorgetti L. Nonlinear control of transcription through enhancer-promoter interactions. Nature. 2022; 604(7906):571–577. 35418676 10.1038/s41586-022-04570-y PMC9021019

[4] Moore JE , Purcaro MJ, Pratt HE et al. Expanded encyclopaedias of DNA elements in the human and mouse genomes. Nature. 2020; 583:699–710. 32728249 10.1038/s41586-020-2493-4 PMC7410828

[5] The GTEx consortium atlas of genetic regulatory effects across human tissues. Science. 2020; 369:6509, 1318–1330. 32913098 10.1126/science.aaz1776 PMC7737656

[6] Gupta RM , Hadaya J, Trehan A, Zekavat SM, Roselli C, Klarin D, Emdin CA, Hilvering CRE, Bianchi V, Mueller C, Khera AV, Ryan RJH, Engreitz JM, Issner R, Shoresh N, Epstein CB, de Laat W, Brown JD, Schnabel RB, Bernstein BE, Kathiresan S. A Genetic Variant Associated with Five Vascular Diseases Is a Distal Regulator of Endothelin-1 Gene Expression. Cell. 2017; 170(3):522–533. e15. 28753427 10.1016/j.cell.2017.06.049 PMC5785707

[7] Williams AM , Jensen DM, Pan X, Liu P, Liu J, Huls S, Regner KR, Iczkowski KA, Wang F, Li J, Gallan AJ, Wang T, Baker MA, Liu Y, Lalehzari N, Liang M. Histologically resolved small RNA maps in primary focal segmental glomerulosclerosis indicate progressive changes within glomerular and tubulointerstitial regions. Kidney Int. 2022; 101(4):766–778. 35114200 10.1016/j.kint.2021.12.030 PMC8940673

[8] Mishra MK , Liang EY, Geurts AM, Auer PWL, Liu P, Rao S, Greene AS, Liang M, Liu Y. Comparative and Functional Genomic Resource for Mechanistic Studies of Human Blood Pressure-Associated Single Nucleotide Polymorphisms. Hypertension. 2020; 75(3):859–868. 31902252 10.1161/HYPERTENSIONAHA.119.14109 PMC7035167

